# PMMA-Based Composite Gel Polymer Electrolyte with Plastic Crystal Adopted for High-Performance Solid ECDs

**DOI:** 10.3390/polym15143008

**Published:** 2023-07-11

**Authors:** Zhou Zhou, Yongkang Tang, Gang Li, Gang Xu, Yong Liu, Gaorong Han

**Affiliations:** 1 School of Materials Science and Engineering, Zhejiang University, Hangzhou 310058, China; 22126087@zju.edu.cn (Z.Z.);; 2 CNBM Bengbu Design & Research Institute for Glass Industry Co., Ltd., Bengbu 233000, China; 3 Shanxi-Zheda Institute of Advanced Materials and Chemical Engineering, Taiyuan 030001, China

**Keywords:** succinonitrile, plastic crystal electrolyte, electrochromic devices, transparent electrolyte

## Abstract

A PMMA-based gel polymer electrolyte (GPE) modified by a plastic crystal succinonitrile (SN) was synthesized using a facile solvent-casting method. The effects of SN additives upon lithium-ion dissociation and ionic conductivity were investigated primarily using Fourier transform infrared spectroscopy and electrochemical impedance spectroscopy, accompanied by other structural characterization methods. The results show that SN is distributed uniformly in the PMMA matrix with a high content and produces vast dipoles that benefit the dissociation of lithium salt. Hence, the SN-modified GPE (SN-GPE) achieves an excellent ionic conductivity of 2.02 mS·cm^−1^ and good mechanical properties. The quasi-solid-state ECD fabricated using the SN-GPE exhibits stable cyclability and excellent electrochromic performance, in which the bleaching/coloration response time is 10 s/30 s. These results add significant insight into understanding the inter- and intra-molecular interaction in SN-GPEs and provide a type of practicable high-performance GPE material for solid electrochromic devices.

## 1. Introduction

Electrolytes have been recognized as the essential constituent in the operation of electrochemical devices, for example, flexible electronic devices [1,2,3,4], battery systems [5,6,7,8,9,10], electrochemical capacitors [11,12], and electrochromic devices (ECDs) [13,14]. For the advantages of soft tenacity, no risk of leakage, and intimate contact with electrodes, the gel polymer electrolyte (GPE), typically known as a compound system wherein the liquid electrolyte phase is well-retained in the polymer matrix, has been regarded as one of the most promising electrolytes. However, its undesirable low cycle stability due to the high degree of liquid electrolyte wettability and unsatisfactory ion conductivity discourages the practical usage of the GPE [15,16]. Due to the combined merits of good conductivity from the lithium-conducting percolation network [17] and ceramic–polymer interfaces [18], along with high mechanical strength and electrochemical stability, introducing inorganic particles into the polymer system is one of the current research’s foci [19]. Wang et al. [20] used the ice-templating method to build a vertically aligned ceramic/polymer composite electrolyte. The vertical ion-conducting path conducted by the active filler Li_1.5_Al_0.5_Ge_1.5_(PO_4_)_3_ (LAGP) assured fast ion transport, reaching a conductivity of 1.67 × 10^−4^ S/cm at room temperature. Though inorganic fillers are considered promising candidates for electrolyte additives, excessive surface energy still causes particle aggregation and phase separation between the additives and the polymer matrix, thus significantly weakening the transparency of the electrolyte and limiting its application in the field of ECDs.

Succinonitrile (SN), one of the prospective unique functional fillers, is known as a typical molecular plastic crystal that may exhibit short-rang crystal rotation and orientation disorder, while the average molecular centers of mass define the long-range ordering nature of the crystal lattice [21]. Maier et al. [22] introduced succinonitrile into PEO and P(VDF-HFP)-based polymer electrolytes as an additive for the combination of enhanced mechanical and electrical properties, which works as a solvent for lithium salt and could decrease the crystallinity of the polymer matrix. Luo et al. [23] propose an in situ cross-linked succinonitrile-based electrolyte with an optimized composition design. The concentrated coordination structure takes advantage of succinonitrile, leading to an improved electrochemical window and stable electrode–electrolyte interface. However, the research mentioned above demands rather complicated and time-consuming manufacturing processes. Wang et al. [24] synthesized a highly transparent GPE with poly(methyl methacrylate) (PMMA) and succinonitrile via a simple blending method, and the enhanced ion transport characteristics successfully ensure its application to the assembly of electrochromic devices (ECDs). The study not only provides a facile way to prepare SN-adopted gel electrolytes but also demonstrates that the ratio between SN and 1,2-propanediolcyclic carbonate (PC) strongly affects the ionic conductivity of the GPE. Unfortunately, the in-depth understanding of the ion transport mechanisms and the interaction between the polymer matrix and fillers/plasticizers is not clear yet, which plays a critical role in the optimization formula control, thus affecting the electrochemical or mechanical properties.

The present work aims to understand the related effect of SN additives on the structural and electrochemical performance of SN-adopted PMMA-based GPEs (SN-GPEs), which were synthesized using the solvent casting method. The structure and property characterization of SN-GPEs was conducted to investigate the heterogeneous effects upon Li^+^ ions transport mechanisms, especially the intra- and inter-molecular interactions between the functional groups. Based on the above research findings, the optimal proportion of SN-GPEs concerning the electrochromic performances has been preliminarily determined, reaching an excellent ionic conductivity of 2.02 mS·cm^−1^ and high transmittance in the visible light range.

## 2. Experimental Details

**Materials:** Succinonitrile (SN, purity > 99%), lithium perchlorate (LiClO_4_, purity > 99.9%), 1,2-propanediolcyclic carbonate (PC, purity > 99.7%), and poly(methyl methacrylate) (PMMA, heat stability and optics grade) were purchased from Macklin. Acetone was purchased from Sinopharm Chemical Reagent Co. Ltd. (Shanghai, China). PMMA was dried in the oven at 60 °C, LiClO_4_ was dried in a vacuum oven at 100 °C for 24 h before use, and other chemical agents were used without further purification.

**Preparation of SN-GPE Membranes:** Firstly, the measured amount of SN (0 mL for SN-0, and 2.3 mL, 4.7 mL, 9.5 mL, and 19 mL for SN-1~4, respectively) was added into 5 mL PC drop by drop, and the mixture was stirred at room temperature for 1 h. Then, the dried LiClO_4_ was added to the SN/PC mixture to form an electrolytic solution and stirred for another 4 h. Next, the concentration of LiClO_4_ respective to PC was fixed at 0.5 M. A total of 2.6 g PMMA was dissolved in 5 mL acetone with a vigorous stir for 6 h at room temperature. The electrolytic solution was then slowly added to the PMMA solution, and the mixture was stirred at room temperature for 3 h. The final transparent sol precursors, with the nominal value of SN content varying from 20 wt% to 65 wt%, were poured onto a releasing paper and dried in a vacuum oven at 50 °C for 48 h to evaporate the acetone, then the gel polymer electrolyte (GPE) membrane was obtained. The film thickness with different SN fillers was managed to be around 1.0 mm ± 0.1 mm. The GPE samples fabricated with the SN:PC molar ratio of 0.5:1, 1:1, 2:1, and 4:1 were labeled SN-1, SN-2, SN-3, and SN-4, respectively. The control group, GPE without SN, was synthesized and similarly labeled SN-0. It is worth noting that the electrolyte membranes SN-3 and SN-4 separated a certain amount of SN and PC during evaporation, possibly raised by the limited capacity threshold of SN in the PMMA matrix. The separated SN/PC solution is denoted as the “unabsorbed solution” and will be discussed later.

**Characterization**: X-ray diffraction (XRD, LabX XRD-6000, SHIMADZU) data were collected at room temperature over the range of 5° ≤ 2θ ≤ 80° at a scanning rate 4° min^−1^ and step length of 0.02° with Cu Kα radiation. Scanning electron microscopy (SEM, S-3400 I, Hitachi) was used to characterize the samples’ morphology with an accelerating voltage of 5.00 kV, magnification of ×1000, and a working distance of 8.2 mm. The intra- and inter-molecular interactions were analyzed using Fourier transform infrared spectroscopy (FTIR, Nicolet iS50, Thermo Fisher, Macquarie Park, Australia) in the range of 400 cm^−1^ ~ 4000 cm^−1^. The electrochemical impedance spectroscopy (EIS) was carried out to obtain the ionic conductivity using an electrochemical workstation (CHI 660E, Shanghai, Chenhua) in the frequency between 0.1 Hz and 1 MHz on a bias of 5 mV. A homemade ion-blocking electrode with the gel electrolyte assembled between two stainless steel plates was employed in the EIS tests. The circumference was separated by a Teflon sleeve to avoid direct contact between the two stainless electrodes. In this test method, the interfacial reaction between lithium ions and the electrode was not involved, and therefore, it is also called a blocking electrode. The detailed manufacturing process is stated in our previous work [19]. The direct current (DC) potential static polarization method was carried out to measure the ion transference number under the potential of 1 V using the same electrochemical workstation and testing devices mentioned above. The optical spectra of ECDs were measured using an ultraviolet-visible spectrophotometer (UV-Vis, Cary 5000, Agilent) over the 300 nm~900 nm wavelength range. The mechanical properties of GPEs were characterized at room temperature using dynamic mechanical analysis (DMA, TAQ800, TA). The GPE samples with a dimension of 30 mm × 8 mm × 2 mm (length × width × depth) were employed to obtain the stress–strain curves in a tensile mode.

## 3. Results and Discussion

**Figure 1:**
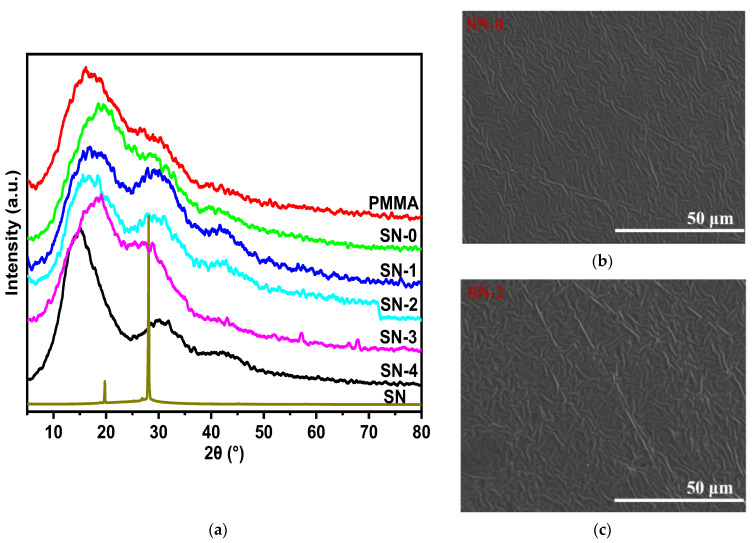
(**a**) XRD diffraction of different SN-GPE samples and PMMA; SEM images of the surface morphology of (**b**) SN-0 and (**c**) SN-2 samples.

The XRD profiles of the SN-GPE samples are shown in Figure 1a, accompanied by the results of PMMA and SN. The three broad bands at about 2θ = 15°, 30°, and 42° of PMMA illustrate a typical feature of the amorphous polymer [25], and the sharp characteristic peaks at 2θ = 20° and 28° are, respectively, attributed to (110) and (200) planes of neat SN [26]. One can find no diffraction peak, but broad bands in the SN-GPE samples indicate that PMMA dominates the structure of the SN-GPE. All SN-GPE samples exhibit flat and uniform surface morphologies with a few wrinkles (see Figure S1), and two representative samples of SN-0 and SN-2 are shown in Figure 1b,c. The SEM results presented confirm that all the samples remain amorphous, which is in correspondence with the XRD results.

**Figure 2:**
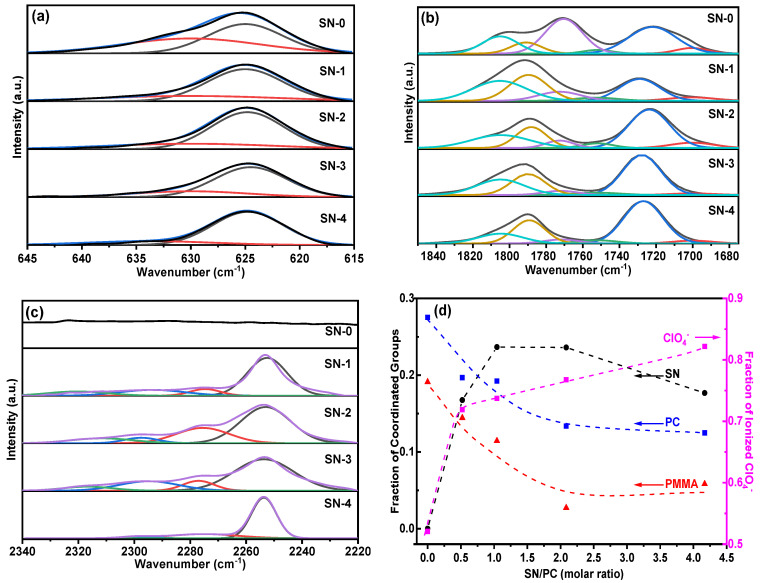
FTIR spectra corresponding to (**a**) ClO_4_^−^, (**b**) carbonyl group, and (**c**) C≡N vibrational modes in SN−GPEs. (**d**) The fractions of spectroscopically ionized ClO_4_^−^, coordinated PC, coordinated PMMA, and coordinated SN in SN−GPE versus SN:PC molar ratio are calculated based on the FTIR spectra.

High-quality FTIR spectra were acquired to investigate the ion-conducting mechanism in the SN-GPE system (see Figure S2a), and the magnified specific bands were analyzed in detail. As shown in Figure 2a, the infrared spectra in the range of 615 cm^−1^~645 cm^−1^ relate to the vibrational mode of ClO_4_^−^. Based on the Gauss fitting, two contributions with the maxima at ~624 cm^−1^ and 630~635 cm^−1^ could be found. The band at the high wavenumber region corresponds to the “spectroscopically free” ClO_4_^−^ vibrational mode, while the band at the low wavenumber region corresponds to the vibrational mode of the contact species ClO_4_^−^ [27]. By integrating the peak areas, the fraction of “free” ClO_4_^−^ could be calculated by the ratio of the “free” ClO_4_^−^ area and total area, as shown in Figure 2d, indicative of an increased behavior with the amount of the SN:PC ratio. As the degree of “free” ClO_4_^−^ could represent the fraction of free Li^+^ ions, one can learn that as the SN:PC ratio increases, the proportion of free Li^+^ in the electrolyte increases gradually.

The infrared spectra in the range of 1680 cm^−1^~1840 cm^−1^, as shown in Figure 2b, are contributed by the absorption peaks of the carbonyl groups, in which the peaks in the vicinity of about 1790 cm^−1^ and 1725 cm^−1^ correspond to the carbonyl groups of PC and PMMA, respectively. For pure PC, the IR spectrum contains two peaks in the carbonyl region, the C=O stretching band at about 1805 cm^−1^, and a Fermi resonance at about 1790 cm^−1^ [28]. After the coordination of the carbonyl group with Li^+^, both peaks shift towards a lower IR region at about 1772 cm^−1^ and 1752 cm^−1^. Therefore, the relative amount of the coordinated carbonyl groups in PC can be determined by calculating the proportion value between the coordinated and total peak areas [28], as shown in Figure 2d, exhibiting a monotonic decrease with the increased SN:PC ratio. For PMMA, two peaks can be identified with Gauss fitting in the IR spectrum range of 1680 cm^−1^~1760 cm^−1^. The peak at about 1729 cm^−1^ represents the free carbonyl group of PMMA, while the shoulder peak at about 1700 cm^−1^ corresponds to the carbonyl groups coordinated with Li^+^ [29]. Then, the fraction of the coordinated carbonyl groups in PMMA could be calculated in the same way as mentioned above for PC, and its SN:PC ratio dependence shows a similar decrease trend to PC, as shown in Figure 2d.

The IR spectra corresponding to SN’s C≡N stretch mode are shown in Figure 2c, where SN-0 shows a flat curve between 2220 cm^−1^ and 2340 cm^−1^. According to the previous report [30], the peak at a low wavenumber could be assigned to the C≡N of SN, which consists of the band at 2253 cm^−1^ corresponding to the stretch mode of pure SN (see Figure S2a) and the band at 2276 cm^−1^ corresponding to the coordinated one with Li^+^. The bands at a high wavenumber are assigned to the combination of the C-C stretch at 918 cm^−1^ and CH_3_ deformation at 1375 cm^−1^ [31], and their shift modes correspond to the coordination between SN and Li^+^ cations. As the last bands are not directly correlated with the nitrile group, the fraction of the coordinated C≡N group with Li^+^ in SN can be calculated based on the area ratio of the bands at 2253 cm^−1^ and 2276 cm^−1^, as shown in Figure 2d, showing a contrary trend compared with that of the coordinated PC and PMMA, which is the fraction of the coordinated SN increase with the SN:PC ratio. Therefore, it can be concluded that the coordination ability with Li^+^ of the C≡N groups in SN is stronger than that of the C=O groups in PC/PMMA, which comes from the high polarity of the C≡N groups.

Further analysis was conducted on the FTIR spectrum of the “unabsorbed solution” (see Figure S2a), exhibiting the characteristic absorption bands at ~1785 cm^−1^ for the carbonyl group from PC, at ~2253 cm^−1^ for the nitrile group from SN, and at ~624 cm^−1^ for ClO_4_^−^ from LiClO_4_. The absence of the C=O peak belonging to PMMA declares that the unabsorbed solution consists of only SN/PC and LiClO_4_. This result indicates that owing to the limited capacity threshold of the PMMA matrix, the further addition of SN would cause the separation phenomenon accompanied by a considerable amount of PC and LiClO_4_ solution out of PMMA. Excessive SN tends to form dense aggregates holding PC and lithium salt solution, separating from the SN-GPE system, and reducing the ion transport pathways provided by SN. It could be reasonable to believe that though the residual lithium salt exhibits a higher ionized fraction, the ionic conductivity and ionic transference number would be obstructed by the lower absolute amount of free Li^+^ ions and rigid polymer chains.

**Figure 3:**
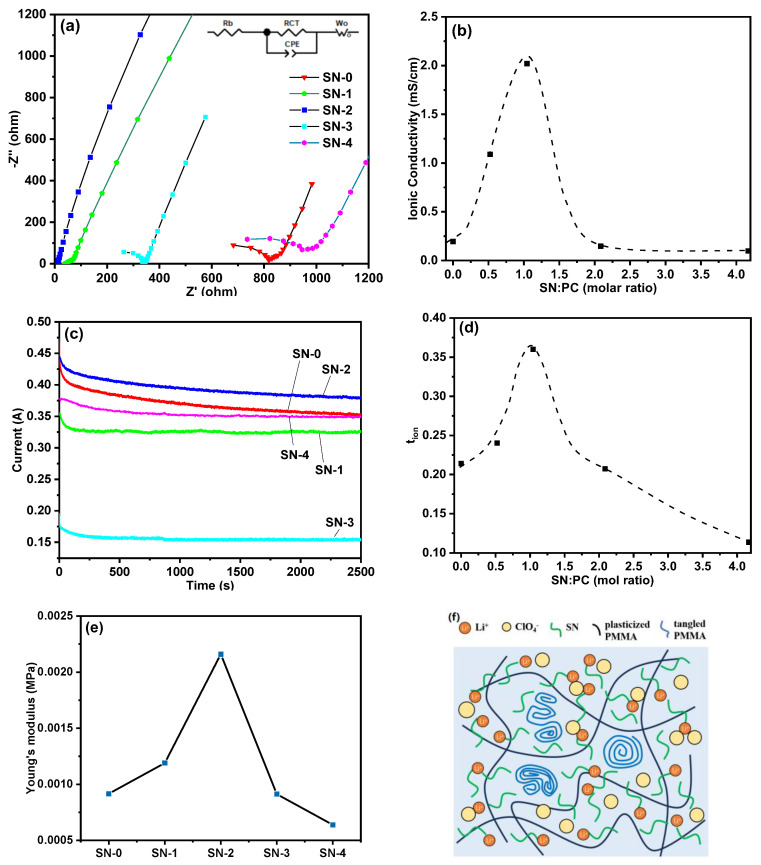
(**a**) Nyquist plots of the SN−GPE samples. (**b**) SN:PC molar ratio dependence of ion conductivity. (**c**) Chronoamperometry plots of the SN−GPE samples. (**d**) SN:PC molar ratio dependence of the ionic transference number (*t_ion_*). (**e**) SN:PC molar ratio dependence of Young’s modulus. (**f**) Schematic diagram of PMMA-based composite gel polymer electrolyte with SN adopted.

The ionic conductivities of SN-GPEs were measured using electrochemical impedance spectroscopy (EIS), and the Nyquist plots are shown in Figure 3a, which are generally divided into two regions: a semicircle at high frequency and a straight line at low frequency. The points and lines in Figure 3 represent the measured and fitted data, respectively. The ion conductivity (*σ*) is governed by the following equation [19]:

(1) $$\sigma =\frac{d}{R_{b}\cdot A}$$

where *R_b_* is the bulk resistance, *A* is the area of the stainless steel electrode, and *d* is the thickness of the sample. The EIS plots are fitted according to the model (see Figure S6), and the fitted data for all circuit elements are shown in Table S1. The calculated ionic conductivity of the films, summarized in Figure 3b, increases almost exponentially with the SN:PC ratio until the ratio value reaches about 1.0, proving that the addition of SN plays a significant role in the increase in ion conductivity, which could be attributed to the addition of plastic crystal SN that blocks the entanglement of the PMMA chains by increasing the number of free segments to enhance the conduction of lithium ions in the polymer [32,33]. Meanwhile, SN additives could also be applied as a carrier for lithium-ion migration [34]. Therefore, the ionic conductivity of SN-2 is relatively high at 2.02 mS·cm^−1^. When the ratio value exceeds 1.0, the polymer cannot hold enough SN/PC mixture, and the extra SN additives separate the PC and lithium salt solution from the PMMA matrix, resulting in the decline in ionic conductivity in SN-3 and SN-4. The role of the “unabsorbed solution” is of great significance in the ion dissociation and transport process; SN/PC plays a critical role in this process as the SN and PC molecules are supposed to have high-polarity groups to facilitate the dissociation of lithium salts as well act as a good carrier of Li^+^.

The ionic transference number (*t_ion_*) of the SN-GPE samples was measured using the DC polarization method, as shown in Figure 3c,d, which is governed by the following equation [35]:

(2) $$t_{ion}=\frac{I_{i}-I_{f}}{I_{i}}$$

where *I_i_* and *I_f_* are the initial and final steady currents, respectively. From Figure 3c, the time dependence of all samples’ current gradually decreases from the initial state to the steady state owing to the ionic species depletion in the electrolyte, and the steady current relates to the polarized electrolyte and the electron migration in the blocking electrode. As shown in Figure 3d, the *t_ion_* of SN-0 is 0.20, which is in correspondence with the previous report [36]. The value then first increases with the SN:PC ratio; after the SN:PC ratio value reaches 1.0, *t_ion_* then goes down, which is very similar to the ionic conductivity. At higher SN loadings, the excessive SN/PC mixture will be squeezed out in SN-3 and SN-4 due to the limited capability of the PMMA matrix, resulting in a decreased Li^+^ amount in the membranes. The SN-2 sample has the best contact between the electrolyte and the electrode, which ascends the ionic conductivity significantly, as revealed in the last section, and benefits practical application in the solid ECD.

As shown in Figure 3e, Young’s modulus calculated from the strain–stress curves (see Figure S3 DMA tests) increases with the SN:PC ratio at first, which could be attributed to the strengthening effect of the SN additives. The value exhibits a maximum at SN-2, after which Young’s modulus decreases because the SN additives separate the membranes.

Compared with other inorganic or organic–inorganic compound fillers, as shown in Table 1, the above results demonstrate the superiority of SN as a filler in transparent electrolyte membranes. The structure of the SN-GPE is schematically shown in Figure 3f. The SN fillers are supposed to serve as transportation channels for lithium ions, contributing to increased ionic conductivity [37], and provide GPEs with enhanced mechanical strength and dimensional stability like other inorganic or compound fillers. Apart from that, SN fillers show good compatibility with the gel polymer, confirmed by the FTIR tests (see Figure S2b) [38]. Therefore, a PMMA-based gel polymer could hold an extremely high SN filler content (~36 wt% in nominal value), maintaining good formability, heterogeneity, and transparency. In contrast, the contents of the inorganic filler are limited due to their excessive surface energy and difficulty in forming transparent membranes.

**Figure 4:**
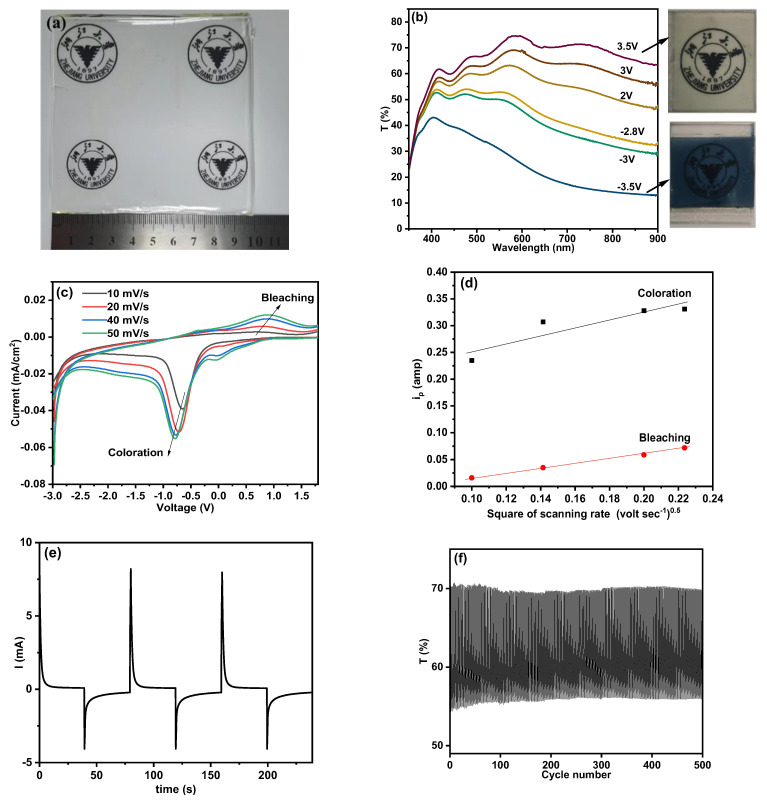
(**a**) Digital image of the SN−GPE sample (SN−2). (**b**) Transmittance of FTO/WO_3_/SN−2/FTO ECD at different voltages. (**c**) CV curves of FTO/WO_3_/SN−2/FTO ECD at various scan rates. (**d**) Coloration/bleaching peak current density as a function of the square root of the scanning rate. (**e**) Current versus time for electrochromic switching of ECD (based on CA). (**f**) Transmittance at 633 nm of ECD over 500 cycles (based on CA).

A representative digital image of an SN-GPE (SN-2) is shown in Figure 4a. Based on a membrane with a thickness of 0.3 mm as the electrolyte, a prototype of the half device was assembled using WO_3_-coated FTO glass as the cathode and another FTO glass as the anode (See Figure S4). The voltage applied to the device causes lithium ions in the electrolyte layer to move along the electric field, while the electrons move in the opposite direction. The movement of ions (electrons) into the electrochromic (ion storage) layers are responsible for the coloration (bleaching) of the ECDs [24].

WO_3_ + xLi^+^ + xe^−^ ↔ Li_x_WO_3_ transparent           dark blue(3)

To further illustrate the bleaching/coloration state of the ECD, the in situ transmittances of the device at various voltages are shown in Figure 4b, accompanied by the digital images of the ECD at the bleaching and coloration states. The CVs of the ECD were carried out between −2.8 V and 2.8 V. The cell voltage recorded is the potential difference of WO_3_ against the FTO counter electrode. When a positive voltage was applied to the ECD, it remained colorless at the bleached state, indicating that the cathodically coloring electrode was oxidized to WO_3_. While applying a negative voltage (0 V to −2.8 V) to the ECD, the color changed to blue resulting from the reduction in WO_3_. The optical modulation at 633 nm is about 48%, corresponding to the bleaching state at 3.5 V with a transmittance value of 70% and the coloration state at −3.5 V with a transmittance value of 22%. The dark blue appearance of the ECD in the coloration state exhibited in Figure 4b demonstrates an intimate contact between the gel electrolyte and electrodes as well as the uniform distribution of lithium ions in the system that helps transport lithium ions homogeneously into the WO_3_ layer resembling the behavior of liquid electrolyte. To investigate the interface between WO_3_/FTO and electrolyte, the CV tests with various scanning rates were conducted upon the ECD, as shown in Figure 4c. The effective diffusion coefficients are calculated according to the Randles–Sevcik equation [43] as follows:

(4) $$I_{p}=2.69\times 10^{5}n^{\frac{3}{2}}AD_{{Li}^{+}}^{\frac{1}{2}}\nu^{\frac{1}{2}}C_{{Li}^{+}}$$

where *I*_p_ is the peak current, *n* is the number of electrons in the reaction (*n* = 1), *A* is the area of electrodes in the electrolyte (*A* ≈ 6 cm^2^), $D_{{Li}^{+}}$ is the effective diffusion coefficient of Li^+^ ions, ν is the scan rate, and $C_{{Li}^{+}}$ is the concentration of lithium ions ($C_{{Li}^{+}}$ = 0.5 × 10^−3^ mol mL^−1^). The current peak densities corresponding to the bleaching and coloration reaction were collected and plotted vs. $\nu^{\frac{1}{2}}$, as shown in Figure 4d. The calculated *D* values for bleaching and coloration are 3.03 × 10^−13^ cm^2^s^−1^ and 8.16 × 10^−13^ cm^2^s^−1^, respectively. Figure 4e presents the chronoamperometry curves for the ECD under the potential range of −2.8 V~2.8 V. The response time, defined as the time required to reach 90% of the peak/valley value, is 10 s and 30 s for the bleaching and coloration states. To obtain the cyclic stability of this system, the proper cell voltage for this device was set as −2.8 V~2.8 V based on the CA test. The dynamic transmittance responses of the ECD, collected at 633 nm, are presented in Figure 4f. Though the transmittances of both bleaching and coloration states decrease with the cycle number, the optical modulation still keeps at 14% after 500 cycles (16% at the initial), showing a quite stable switching property. This result further confirms that SN-adopted PMMA-based electrolytes exhibit fine ion-conducting properties and could be considered a potential candidate for ECDs and a hopeful replacement for liquid electrolytes.

## 4. Conclusions

To investigate the modified mechanism of SN plastic crystal as an additive in the electrochemical properties of a PMMA-based gel polymer electrolyte (GPE), a series of SN-modified PMMA-based GPEs were prepared using the solvent casting method and characterized for their structural and electrochromic properties. The results indicate that SN additives could plasticize the PMMA matrix, contribute to the ion solvation process, and provide transport pathways for lithium ions. Additionally, SN in the system can change the Li^+^ distributions from PC molecules and PMMA chains to SN additives, as the high polarity of the nitrile groups inclines to a better binding ability than the carbonyl groups in PC and PMMA. Apart from this, PMMA shows a good absorption capability with SN at as high as around 36 wt%, mainly due to the intermolecular interaction, which contributes greatly to the mechanical and electrochemical properties of the SN-GPE. However, there still exists a limitation of the PMMA matrix to contain SN additives, causing overdosed SN additives to separate out of the film with PC molecules in the state of the solution and suppress ion conductivity.

The SN-GPE with the best match of ingredients exhibits an ion conductivity of 2.02 mS·cm^−1^. The ECD fabricated with the optimized SN-GPE exhibits an excellent electrochromic performance with a high optical modulation of 48% at 633 nm and a 10 s/30 s response time for bleaching/coloration. The results above suggest that the SN-GPE is a potential candidate in the field of quasi-solid-state ECDs. Its electrochemical stability and high transparency suggest this material system is also promising for multiple areas like future high-performance flexible devices and smart windows after exploring the service performance under high-temperature circumstances.

## Figures and Tables

**Table 1:** Comparison of the composite GPEs.

Matrix	Salt/Fillers	Ionic Conductivity (S·cm^−1^)	Optic	Ref.
PEO	LiTFSI/Li_1.5_Al_0.5_Ge_1.5_(PO_4_)_3_	1.67 × 10^−4^	opaque	[20]
PVdF-HFP	LiN(CF_3_SO_2_)_2_/AlO[OH]_n_	~10^−4^	-	[39]
PVdF-HFP	LiTFSI/graphene oxide	1.3 × 10^−3^	opaque	[40]
PVdF-HFP	LiCF_3_SO_3_/ZrO_2_	3.42 × 10^−3^	opaque	[41]
PMMA	LiPF_6_/MA-POSS*	2.77 × 10^−3^	opaque	[42]
PVdF-HFP	LiClO_4_/SiO_2_-MMA	2.22 × 10^−3^	transparent	[19]
PMMA	LiClO_4_/SN	2.02 × 10^−3^	transparent	this work

## Data Availability

Data available on request due to restrictions eg privacy or ethical.

## Supplementary Materials

The following supporting information can be downloaded at: https://www.mdpi.com/article/10.3390/polym15143008/s1, Figure S1: SEM images of the surface morphology of the SN-GPE samples and PMMA; Figure S2: (a) FTIR spectra of the SN-GPE samples, pure SN, PC, and the unabsorbed solution (b) FTIR spectra corresponding to the coordination between C≡N groups and C=O groups; Figure S3: DMA spectra of (a) SN-0 (b) SN-1 (c) SN-2 (d) SN-3 (e) SN-4; Figure S4: Configuration of FTO/WO_3_/SN-GPE/FTO ECD; Figure S5: Schematic representation of the reparation of the SN-GPE membrane; Table S1: Fitting data for EIS data.
