# Supplementary material for: PMMA-Based Composite Gel Polymer Electrolyte with Plastic Crystal Adopted for High-Performance Solid ECDs
Source: Polymers (Basel). 2023 Jul 11;15(14):3008. doi: 10.3390/polym15143008 (PMC10384775; doi:10.3390/polym15143008)
Supplement: Supplementary file 1 [file polymers-15-03008-s001.zip › polymers-2493819-supplementary.pdf]

Supporting Information

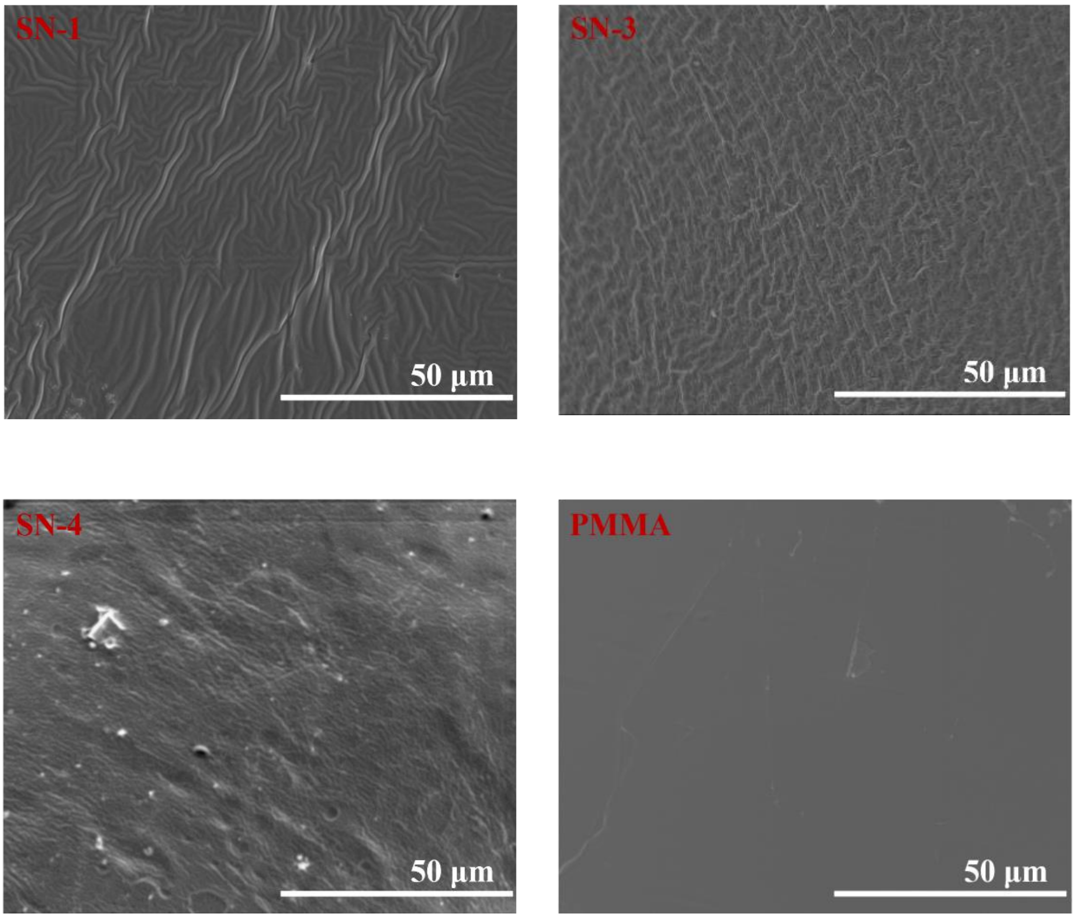

Figure S1. SEM images of the surface morphology of the SN-GPE samples and PMMA. The micrograph shows that the films are all uniform, and adding SN additives diminishes the well-distributed wrinkles.

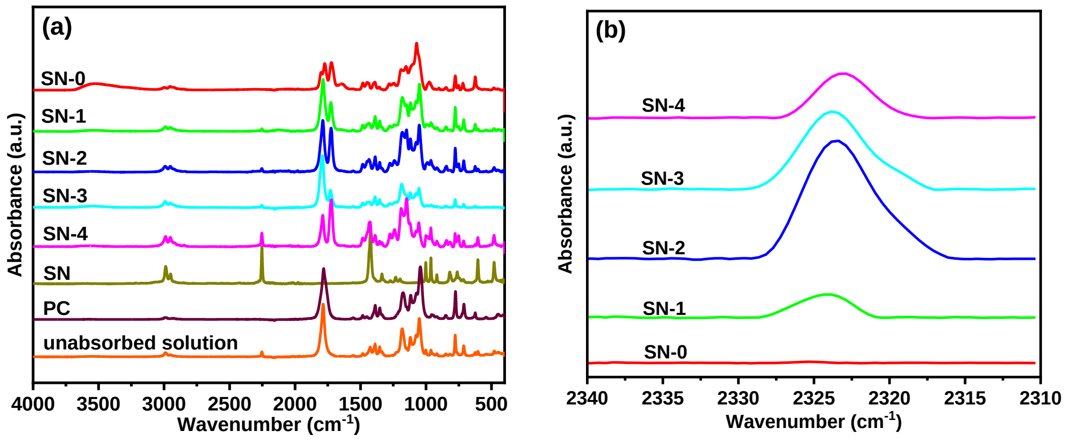

Figure S2. (a) FTIR spectra of the SN-GPE samples, pure SN, PC, and the unabsorbed solution; (b) FTIR spectra corresponding to the coordination between C≡N groups and C=O groups.

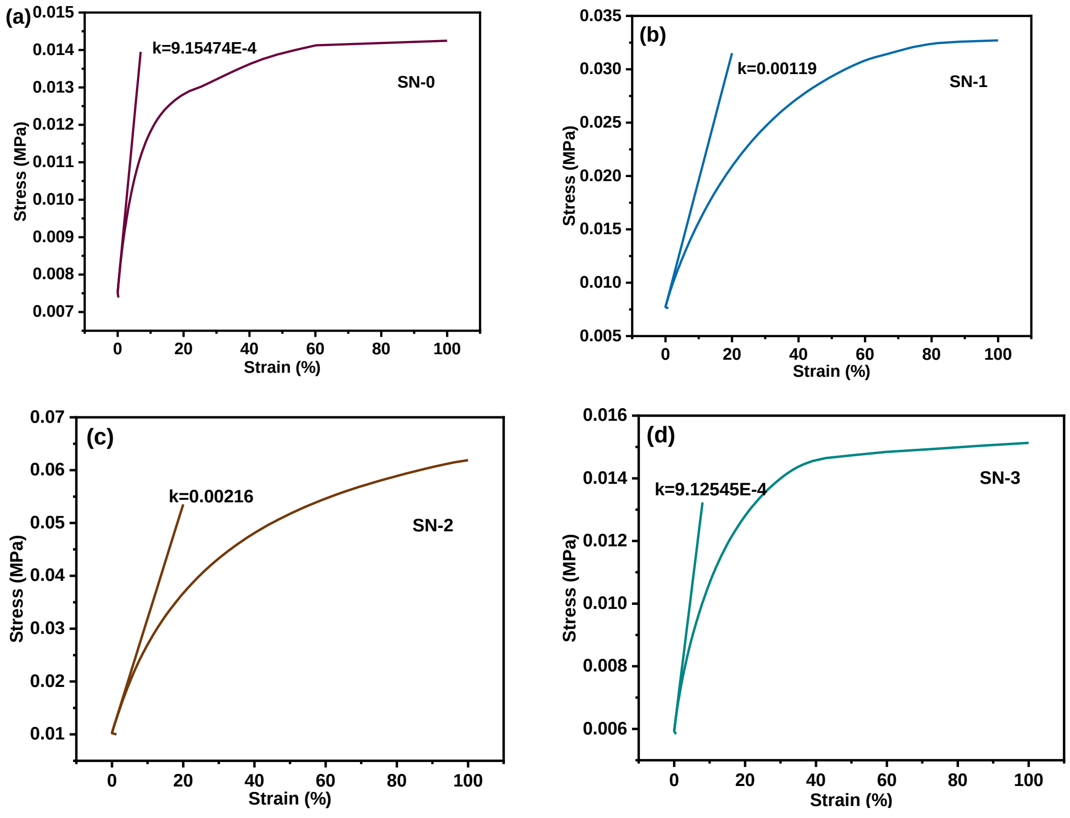

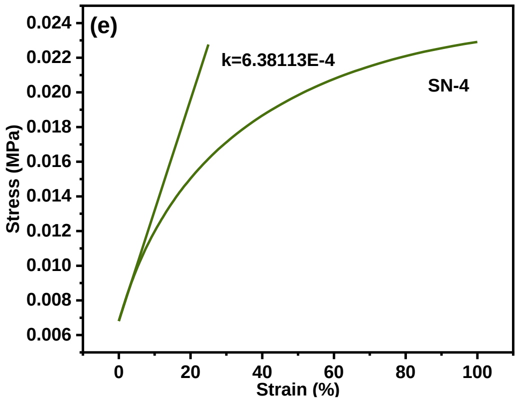

Figure S3. DMA spectra of (a) SN-0; (b) SN-1; (c) SN-2; (d) SN-3; (e) SN-4.

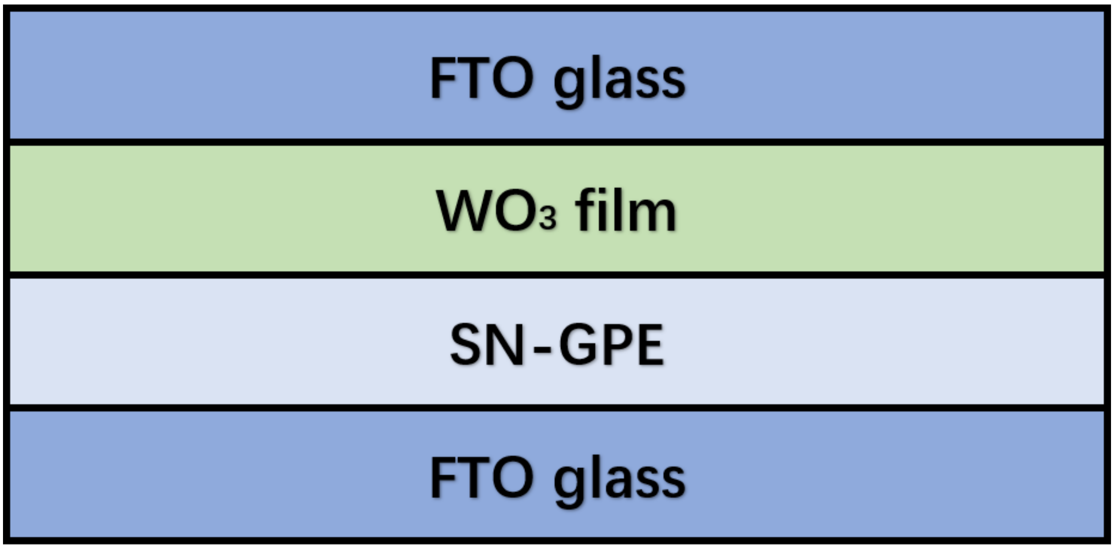

Figure S4. Configuration of $FTO/WO_3/SN$-GPE/FTO ECD.

Fabrication of the $FTO/WO_3/SN$-GPE/FTO ECD: The schematic diagram of the structure of ECD is shown in Fig.S4. The WO_3-coated FTO glass, selected SN-2 membrane, and another FTO glass were stacked and heat-treated at 50ºC for 3 h, then stored in a vacuum box to remove the water. The sandwich-like device was then sealed with a 0.5 cm wide electrically conductive tape (3M Company) and heated at 100 ºC in a vacuum to make the SN-2 film contact closely with the electrodes.

Preparation of the Tungsten oxide film: The 3 × 3 cm^2 glass plates precoated with SnO_2:F (FTO, 15 Ω/square, 3 × 3 cm^2) were used as the substrates. The tungsten oxide films were deposited by reactive direct current magnetron sputtering from a cylindrical rotating tungsten target (500 mm in length, 133 mm in diameter, and 9 mm in thickness with 99.9% purity) in a deposition system based on a vertical continuous high vacuum sputtering pilot production line (FSE-ILSV-RD-450).

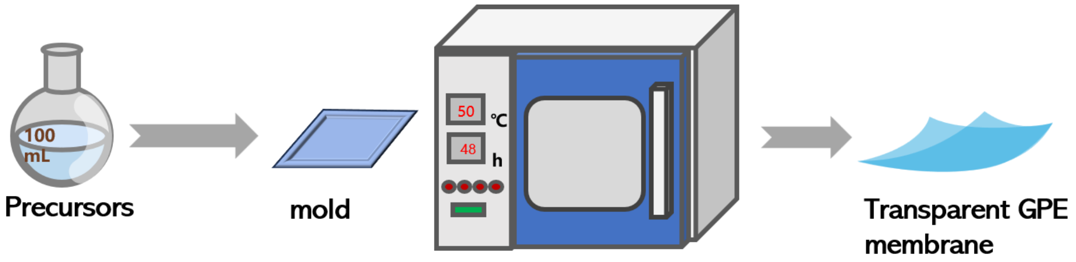

Figure S5. Schematic representation of the reparation of the SN-GPE membrane.

Table S1. Fitting data for EIS data.

Samples	Rb	R_{CT}	CPE-T	CPE-P	W_O-R	W_O-T	W_O-P
SN-0	639.4	176.3	6.45E-10	1.08	187.2	6.03E-4	0.40216
SN-1	35.6	181.8	2.74E-4	0.437	67.06	3.14E-4	0.40002
SN-2	31.99	5.9453	3.13E-4	0.563	0.183	2.72E-7	0.42128
SN-3	229.7	110	1.387E-9	1.055	42355	2.1097E-5	2.15E-4
SN-4	510	875.3	6.467E-10	0.96819	7.953	5.6402E-6	0.38208
